# Analysis of molecular resistance to azole and echinocandin in *Candida* species in patients with vulvovaginal candidiasis

**DOI:** 10.18502/cmm.8.2.10326

**Published:** 2022-06

**Authors:** Ensieh Lotfali, Mahzad Erami, Mahsa Fattahi, Houshang Nemati, Zeinab Ghasemi, Elham Mahdavi

**Affiliations:** 1 Department of Medical Parasitology and Mycology, School of Medicine, Shahid Beheshti University of Medical Sciences, Tehran, Iran; 2 Kashan Shahid Beheshti Hospital, Kashan University of Medical Sciences, Kashan, Iran; 3 Center for Research and Training in Skin Diseases and Leprosy, Tehran University of Medical Sciences, Tehran, Iran; 4 Fertility and Infertility Research Center, Health Technology Institute, Kermanshah University of Medical Sciences, Kermanshah, Iran; 5 Razi Hospital, Tehran University of Medical Science, Tehran, Iran; 6 Department of Medical Parasitology, School of Medicine, Zabol University of Medical Sciences, Zabol, Iran

**Keywords:** Azole, Echinocandin, *Candida* species, Mutations, Vulvovaginal candidiasis

## Abstract

**Background and Purpose:**

Vulvovaginal candidiasis (VVC) is considered the most common mucosal infection caused by *Candida* species. Azoles were considered the first-line treatment for VVC or recurrent vulvovaginal candidiasis (RVVC) in both healthy and immunocompromised populations. Recently, azole-resistant isolates, especially among non-*albicans* *Candida* samples have been encountered. This study aimed to evaluate the antifungal susceptibility profile of *Candida* spp. isolated from VVC or RVVC patients and assess the molecular resistance mechanism of *Candida* spp. to azole and echinocandin.

**Materials and Methods:**

Point mutation analysis was performed on the *ERG11* and *FKS* candidate genes of azole- and caspofungin-resistant *Candida albicans* and *Candida glabrata* isolates. Real-time polymerase chain reaction was performed to gain insight into the differential expression of *ERG11* mRNA.

**Results:**

Variations in the amino acid D116E were observed in fluconazole- and itraconazole-resistant *C. albicans* strains, and changes in amino acid E517Q were observed only in fluconazole-resistant *C. albicans* strains. No polymorphisms were observed in the complete sequence alignment of the *ERG11* gene in one azole-resistant *C. glabrata* isolate. The mutation triggered the changes in the amino acid serine in the reference gene *FKS1* by the leucine at position 642 (S642L) of the isolates.

**Conclusion:**

In patients with persistent or recurrent infection, the choice of an antifungal agent is often challenging and requires monitoring of the antifungal susceptibility of the colonizing strain. *C. albicans* and *C. glabrata* isolates can be resistant to azole and caspofungin antifungal agents without mutations in the *ERG 11* and *HS1* regions of the *FKS1* gene.

## Introduction

Vulvovaginal candidiasis (VVC) is considered the most common mucosal infection caused by *Candida* species [ 1 ]. It is believed that 75% of women experience VVC at least once in their lives [ 2 ]. As is the case with most infections caused by *Candida* spp., *Candida albicans* is isolated from 85-90% of cases [ 3 ]. However, non-*albicans* *Candida* spp. (NACs), which are less sensitive to the frequently administrated topical and oral azole agents, have also increased [ 1 , 3 - 5 ]. In addition, NACs are more frequently involved in recurrent vulvovaginal candidiasis (RVVC), compared to *C. albicans* [ 4 ].

Azoles were considered the first-line therapy for VVC and RVVC in both healthy and immunocompromised populations. Recently, cases of azole-resistant isolates, especially among NACs, have been encountered [ 6 , 7 ]. Mutation in the gene that encodes the 14a-demethylase target, *ERG11*, can contribute to the resistance to azole in *Candida* spp. [ 8 , 9 ]. Alteration in the mRNA expression level of the genes encoding efﬂux pumps *CDR1*, *CDR*, and *MDR* created resistance to azole [ 10 ]. 

In most cases, loss of susceptibility to echinocandins was associated with the alternations in the hot spot region since echinocandins are insignificant substrates for drug efflux transporters [ 11 ]. Loss of susceptibility to echinocandins is facilitated through alternations in the *FKS1* gene, which encodes the extraction of a key element in the cell wall [ 12 ]. The known mutations are associated mainly with hot spot 1 (*HS1*, at positions 641-649) and *HS2* (at positions 1345-1365) regions [ 13 ]. 

The mutations in the *FKS2* gene were found in *C. glabrata* and *C. guilliermondii* strains. Since the loss of *Candida* spp. susceptibility to antifungal treatment has become a major drawback in the management of recurrent infections, the use of other antifungals can be helpful in this regard.

Since various *Candida* spp., even different isolates of specific strains, have different sensitivity to an antifungal agent, it seems that identification of *Candida* at the species level is necessary for the selection of appropriate treatments and avoidance of antifungal failure. The present study aimed to evaluate the antifungal susceptibility profile of *Candida* spp. isolated from patients who were VVC and RVVC-positive. Furthermore, the molecular mechanism of resistance to azole and echinocandin was also assessed.

## Materials and Methods

### Study design

The current investigation was conducted on 75 clinical isolates of vaginal discharge from 147 patients suspected of VVC/RVVC who had referred to the Medical Mycology Laboratory of Razi Hospital, Tehran, Iran in 2019. 

### Sample collection and primary identification

The specimens were gathered using sterile cotton swabs. All specimens were subjected to direct microscopic examination to identify fungal elements. To access pure isolates, all specimens were cultured on CHROMagar *Candida* medium (CHROMagar, France) at 35 °C for 24 h. The specimens were stored in Tryptic Soy Broth (Liofilchem, Italy) at -70 ºC for further analysis.

### DNA extraction

Genomic DNA of each positive isolate was extracted from fresh colony cultures on Sabouraud Dextrose Agar medium (Merck, Germany) at 35 ºC for 24 h using the isopropanol and proteinase K approach based on our previous setup [ 14 ]. The part of the yeast colony was transferred in a 1.5 ml microtube containing DNA extraction buffer and glass beads and vortexed. Subsequently, proteinase K and 20% Sodium dodecyl sulfate were added to it and it was kept warm at 55 ºC for 60 min. Afterward, 6 molar NaCl was added, and the total DNA was precipitated using isopropanol. The precipitate was rinsed in 300 μL ethanol 70%, air dehydrated, rehydrated in 50 μl TE buffer (Merck, Germany), and kept at -20 ºC.

### Polymerase chain reaction amplification of internal transcribed spacers region and Restriction fragment length polymorphism assay

The amplification of the internal transcribed spacers (ITS)1-5.8S-ITS2 region was carried out using the ITS1 (5´-TCC GTA GGT GAA CCT GCG G -3´) and ITS4 (5´- TCC TCC GCT TAT TGA TAT GC- 3´) [ 13 ] with the following program: 95 °C for 15 min; 35 cycles of 30 sec at 95 °C, annealing for 20 sec at 62 °C, 20 sec at 72 °C, and a final extension of 1 min at 72 °C.

The amplicons were seen using electrophoresis on 1.5 % agarose gel in Tris-borate-EDTA buffer (TBE) and DNA stain (Sim Bio, USA). Afterward, all appropriate products were subjected to the *MspI* (Fermentas, USA) restriction enzyme for Restriction Fragment Length Polymorphism (RFLP) assay. Restriction fragments were separated on 2% agarose gel electrophoresis in TBE buffer with DNA safe stain. To ensure precise identification of ambiguous fragments, polymerase chain reaction (PCR) sequencing with ITS primers was carried out by Noorgeen Company.

### Polymerase chain reaction amplification of HWP1 gene

The *HWP1* gene was developed to identify the *C. albicans* complex (i.e, *C. albicans*, *C. africana*, *C. dubliniensis*, *C. stellatoidea*). The PCR using the specific primers *HWP1*-F (5′-TGGTAAGGCGGG ATCGCTT-3′) and *HWP1*-R (5′-GGTCAAAGTTTG AAGATATAC) [ 15 , 16 ] was performed by means of the program: 95 °C for 15 min; 35 cycles of 30 sec at 95 °C, annealing for 20 sec at 60 °C, 20 sec at 72 °C, and a final extension of 1 min at 72 °C.

### In-vitro antifungal susceptibility testing

Itraconazole, miconazole, fluconazole, butoconazole, and caspofungin were obtained from Sigma-Aldrich (St. Louis, MO, USA). The *in-vitro* activity of six antifungal drugs was assessed by means of the Clinical and Laboratory Standards Institute (CLSI) M-60 protocol [ 17 ]. The minimal inhibitory concentration (MIC) of antifungals was determined after 24 h. *Candida parapsilosis* ATCC 22019 was chosen as a control in every run. The results were analyzed using the CLSI M60 guideline.

### Point mutation analysis in ERG11 and FKS candidate genes of azole and caspofungin resistance C. albicans and C. glabrata

The *ERG11* and *FKS* sequencing was performed to determine the possible molecular mechanisms involved in azole and echinocandin resistance. The genomic DNA of azole and echinocandin resistant and susceptible *Candida* isolates was extracted using the former methods. The PCR amplification of the *ERG11* gene was performed by *ERG11* -F- *albicans* (5´- GTT GAA ACT GTC ATT GAT GG-3´) and *ERG11* –R- *albicans* (5´- TCA GAA CAC TGA ATC GAA AG-3´) [ 18 ] ERG-11 F -*glabrata*, (5´-ATGTCCACTG AAAACACT--3´) and *ERG11*- F -*glabrata* (5´-CTAGTACTTTTGTTC TGG-3’) [ 19 ] and *FKS* 1-F -*glabrata* (5´- CCA TTG GGT GGT CTG TTC ACG -3´) and *FKS* 1-R- *glabrata* (5´- GAT TGG GCA AAG AAA GAA ATA CGA C -3´) [ 20 ], and *FKS* 1-F-*albicans* (5´- GAAATCGGCATAT GCTGTGTC -3´) *FKS* 1-R- *albicans* (5´- AATGAACGACCAATGGAG AAG -3´) [ 21 ] primers with the following program: 5 min at 94˚C, 35 cycles for 60 sec at 94 ˚C, 60 sec at 48 ˚C and 90 sec at 72 ˚C. The products were analyzed by electrophoresis on 1% agarose gel in TBE buffer and stained with a safe stain. The products were sequenced and analyzed using the MEGA software (version 7.0.21).

### RNA extraction

Total RNA was isolated from the yeast using the RNX-PLUS kit (Sinaclon, Iran). To remove any DNA pollution, RNA was cured with DNase1 (Fermentas, USA) based on the instructions of the manufacturer. 

### cDNA synthesis

cDNA was synthesized using 3µg RNA, 20 pmoles/µl random hexamer (Fermentas, USA), 20 pmoles/µl Oligo-dT (Fermentas, USA), and 10 mM of dNTP mix (Fermentas, USA). Afterward, it was incubated at 65 °C for 10 min followed by the addition of 20 U Ribolock (Fermentas, USA), 7.5 µl of 5X reverse transcriptase buffer (Fermentas, USA), and 200 U Moloney Murine Leukemia Virus (M-MuLV) reverse transcriptase enzyme (Fermentas, USA). Finally, it was incubated at 70 °C for 10 min followed by incubation at 42 °C for 60 min.

The accuracy of cDNA was checked with Actin gene primers as housekeeping. The PCR condition was an initial denaturation step for 5 min at 94 °C, 30 cycles of denaturation for 30 sec at 94 °C, annealing for 30 sec at 60 °C, an extension for 7 min at 72 °C with a ﬁnal extension of 72 °C.

### Quantitative real-time polymerase chain reaction

After cDNA synthesis, the quantitative real-time PCR was performed. The total volume of PCR reaction mixture )20 µl( included 10 µl Q-Master Mix SYBR Green I (2X) (Ampliqon, Denmark), 0.04µl ROX Dye (50X), 1µl cDNA template, 0.8 µl forward, and 0.8 µl reverse primers (10 pmoles/µl) [ 18 ]. The PCR program was defined as follows: initial denaturation at 94 °C for 10 min, amplification for 35 cycles at 94 °C for 30 sec, 60 °C for 30 sec and 72 °C for 30 sec, and final extension at 72 °C for 5 min. It should be mentioned that all experiments were conducted in triplicate. Ultimately, the information taken from the real-time PCR was analyzed using the REST^©^ software (2009 V2.0.13).

## Results

### Results of initial identification

Only 75 out of 147 specimens were positive for *Candida* spp. Through the use of the chromogenic media, 62 (82.66%) isolates with white to green colony color were identified as *C. albicans*, 11 (14.66%) isolates with pink color were identified as *C. glabrata* and *C. krusei*, and 2 (2.66%) isolates with blue colony known as *C. tropicalis*.

### Results of polymerase chain reaction amplification of internal transcribed spacers region and Restriction fragment length polymorphism assay

**Figure 1:**
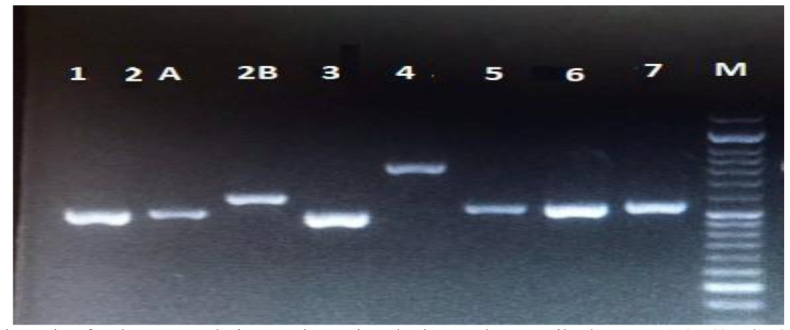
Agarose gel electrophoresis of polymerase chain reaction using the internal transcribed spacers1 (ITS)-F/ITS4-R primers.

**Figure 2:**
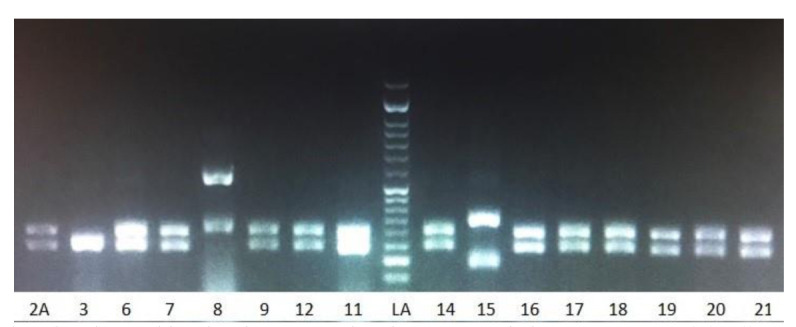
Restriction fragment length polymorphism bands pattern using the *MspI* restriction enzyme: *Candida albicans* (297-238 bp): 2A, 6, 7, 9, 12, 11; *Candida glabrata* (314-557 bp): 8; *Candida krusei* (184-340 bp): 3; *Candida tropicalis* (184-340 bp):15; LA: marker 50 bp.

The amplification of isolates using ITS1-F/ITS4-R was interpreted based on the production of a ~535 bp fragment of *C. albicans*, a ~871 bp *C. glabrata*, a ~510 bp *C. krusei*, and a ~524 bp *C. tropicalis* (Figure 1). The RFLP patterns indicated that *C. albicans* 62 (82.66%) was the predominant causative agent isolated from vaginal discharge followed by *C. krusei* 6 (8%), *C. glabrata* 5 (6.6%), and *C. tropicalis* 2 (2.1%) (Figure 2). 

*Candida albicans* (2A, 5, 6, 7), *Candida tropicalis* (2B), *Candida glabrata* (4), and *Candida krusei* (1, 3); M: marker 100 bp.

### Polymerase chain reaction amplification HWP1 gene

**Figure 3:**
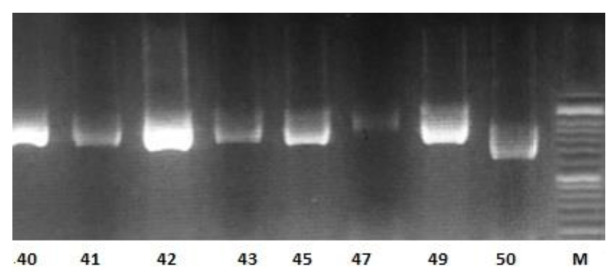
Agarose gel of polymerase chain reaction amplification with *HWP1* gene; *Candida albicans* (~900 bp): 40, 41, 42, 43, 45,47, 49; *Candida africana* (~700 bp): 50; M: marker 100 bp

 The results of amplification of isolates with the *HWP1* gene revealed that among *C. albicans* complex, 56 strains with ~900 bp were categorized as *C. albicans*, and two isolates with ~700 bp were identified as *C. africana* (Figure 3). It should be noted that no *C. dubliniensis* and *C. stellatoidea* isolates were identified.

### Antifungal susceptibility testing

Table 1 presents the antifungal susceptibilities of the *Candida* spp. Three isolates of *C. albicans* were resistant to itraconazole (MIC≥8 µg/mL) and fluconazole (MIC>32µg/mL). All *C. glabrata* isolates were resistant to fluconazole (MIC≥16 µg/mL). Moreover, three strains of itraconazole (MIC≥4 µg/mL) and one isolate showed resistance (MIC 4 µg/mL) to caspofungin. No antifungal resistance was observed in *C. tropicalis* and *C. krusei* to the agents used in this study.

**Table 1:** Antifungal susceptibility of *Candida* spp. using the broth microdilution method M60 (N=75)

Antifungal		*Candida albicans* (62)	*Candida krusei* (6)	*Candida glabrata* (5)	*Candida tropicalis* (2)
Fluconazole	S (%)	59 (95)	NT	-	2 (100)
	SDD (%)	2 (3.22)	NT	-	-
	R (%)	1 (1.61)	NT	5(100)	-
Itraconazole	S (%)	60 (96.7)	6 (100)	2 (40)	2 (100)
	SDD (%)	-	-	-	-
	R (%)	2 (8/3.2)	-	3(60)	-
Butoconazole	S (%)	61 (98)	6 (100)	5 (100)	2 (100)
	I (%)	1 (1.61)	-	-	-
	R (%)	-	-	-	-
Miconazole	S (%)	62 (100)	6 (100)	5 (100)	2 (100)
	I (%)	-	-	-	-
	R (%)	-	-	-	-
Caspofungin	S (%)	59 (95)	6 (100)	4 (80)	2 (100)
	I (%)	-	-	-	-
	R (%)	3 (5)	-	1 (20)	-

SDD: susceptible dose depended, R: resistance, S: susceptible, NT: not tested

### Analysis of amplification of ERG11 and FKS1 genes of C. albicans and C. glabrata

Augmentation of the *ERG11* gene of *C. albicans* strains (two of which were resistant to fluconazole and itraconazole, one only showed loss of susceptibility to fluconazole, and 55 were sensitive to azole) was achieved using PCR. Although multiple silent single nucleotide polymorphisms were detected, missense mutations trigger amino acid alternations were observed in two strains. Changes in amino acid D116E were discovered in fluconazole-and itraconazole-resistant strains. Moreover, changes in amino acid E517Q were detected only in fluconazole-resistant strains. 

No polymorphisms were found in the complete sequence arrangement of the *ERG11* gene of one azole-resistant *C. glabrata* isolate. The piece of 450 and 725 bp of the *FKS1* gene of *C. albicans* (3 caspofungin-resistant and 2 caspofungin-sensitive) and *C. glabrata* (1 caspofungin-resistant and 1 caspofungin-susceptible) strains was subjected to PCR sequencing. 

Nucleotide and protein alignments were performed to determine any amino acid changes. Single missense alternation was identified in all *C. albicans* and *C. glabrata* resistant strains, unlike the sensitive and reference strains. The mutation triggers the changes of serine in the reference *FKS1* gene by the leucine in the isolates at position 642 (S642L). 

### Quantitative real-time PCR amplification

Quantitative real-time PCR was conducted to gain insight into the differential appearance of the *ERG11* mRNA expression level. The expression degree of the target gene was standardized to the expression of Actin as a housekeeping gene, and finally, the data were analyzed by the REST© software (2009V2.0.13).

**Figure 4:**
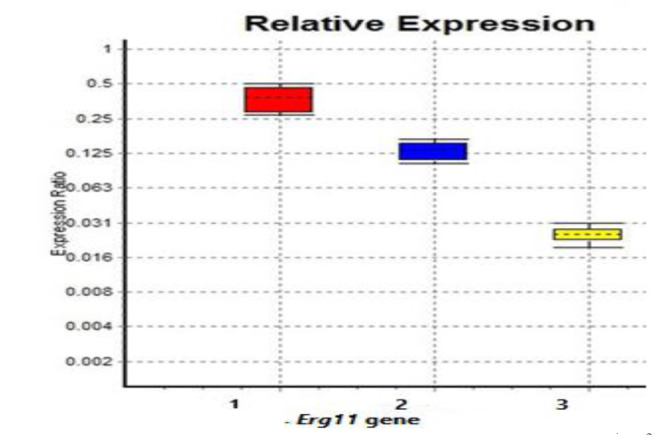
Low expression of *ERG11* mRNA expression level of *Candida albicans*, one *Candida glabrata* resistant isolate, compared to susceptible isolates

The expression of *ERG11* mRNA in terms of resistance was relatively low, compared to the reference strains' expression of three *C. albicans* and one *C. glabrata* isolates (expression ratio: 0.129 up to 0.366-fold changes) (Figure 4).

## Discussion

The obtained results revealed that three strains of *C. albicans* were resistant to itraconazole (MIC≥8µg/mL) and fluconazole (MIC>32µg/mL). Moreover, it was demonstrated that the isolates of *C. glabrata* were also resistant to fluconazole (MIC≥16 µg/mL) and itraconazole (MIC≥4 µg/mL). Therefore, these two drugs are not proper substitute medications for resistant pathogens.

The results of a study conducted by Carillo-Munoz et al. [ 22 ] indicated that terbinafine had excellent efficacy against resistant *C. albicans*, compared to fluconazole. However, greater MIC was found in *C. glabrata* (16 µg/ml), compared to other *Candida* spp. (0.125 µg/ml), and very weak MIC was observed in *in vitro* activity. In the present study, full sensitivity to miconazole and butoconazole was detected in *Candida* spp. (Table 1).

Caspofungin is an antifungal drug used for a wide spectrum of fungal infections, including *Candida* spp. infections [ 23 ]. Pfaller et al. collected *Candida* isolates (n=8197) from 91 therapeutic centers throughout the world and determined the sensitivity of caspofungin in the selected isolates [ 23 ]. In their study, which continued for over 4 years, the susceptibility to caspofungin remained unaffected and was prevented up to 99% (<1 µg/ml annually). In the present study, *C. albicans* (3/62) and *C. glabrata* (1/5) isolates did not show susceptibility to caspofungin (MIC 4 µg/mL), while all *C. kruzei* and *C. tropicalis* strains were sensitive to caspofungin.

Alternations in the *ERG11* gene lead to azole resistance in *Candida* spp. [ 24 ]. More than 140 amino acid replacements have been verified until now, which are mainly located in three *HS* regions; specifically in 105-165, 266-287, and 405-488 amino acid regions [ 25 ]. Some of these mutations were associated with azole resistance [ 26 , 27 ].

In a study conducted by Morio et al., 23 amino acid alternations were reported in 60 out of 73 *C. albicans* strains [ 24 ]. Changes in K143R, R467K, V404I, V509M, and F449V amino acids were detected in fluconazole-resistant isolates, while substitutes for D116E, E226D, and V437I amino acids were identified in both susceptible and resistant isolates. Furthermore, three new amino acid replacements (i.e., F449V, V404I, and V509M) were recognized, two of which were sited outside the third *HS* region [ 24 ].

In addition, four substitutes were determined which included N136Y, Y221H, L276S, and Y555447H. In total, 11 out of the 23 amino acid changes were determined equally in azole-resistant and susceptible isolates. In another study, ERG11 gene mutations were reported in *C. albicans* isolates [ 28 ].

In the present study, the full-length *ERG11* sequence of *C. albicans* strains was expanded to the image of any gene alternations occurring in the *ERG11* gene. Point mutations were commonly observed in the *ERG11* gene of azole-susceptible and azole-resistant strains; however, amino acid changes were observed only in a single azole-resistant strain.

The present data highlighted the role of other possible mechanisms in conferring resistance to azoles, including overexpression of genes encoding efflux transporters, overexpression of the *ERG11* gene, and modification in the ergosterol biosynthetic pathway. According to the QRT results, the expression level of the *ERG11* mRNA in three resistant *C. albicans* and two resistant *C. glabrata* isolates was upregulated (0.129 up to 0.366-fold changes), compared to the reference strains.

Point mutations into the *FKS1* gene encoding 1, 3-β-glucan synthase were determined in echinocandins-resistant *Candida* spp. [ 12 ]. These mutations were mainly located in 641 to 649 *HS1* positions, including F641, L642, T643, S645, R647, D648, and P649 mutations, as well as in amino acid regions 1345-1365 (*HS2*), including the R1352 mutation [ 17 , 29 ]. 

In another study, resistance to caspofungin in 85 isolates of *C. albicans* strains has been caused by mutations in the FKS1 gene [ 30 ]. However, in the present study, no mutations were observed in four *C. albicans* isolates that were resistant and susceptible to caspofungin, and only the substitution of polar amino acid serine with non-polar amino acid leucine (S642L) in the *FKS1* gene was detected in the tested isolates. The substitution of serine with leucine in *C. albicans* isolates has been recorded as well Therefore, a mutation in the anticodon region led to the identification of the CUG codon with the seryl and leucyl-tRNA synthetases [ 31 ]. In the present study, the substitution of TCG with TTG led to a missense mutation.

## Conclusion

The obtained results suggest that proper attention should be paid to the antifungal history of the individuals and future requirements for treatment when deciding about the best antifungal drugs for the treatment of VVC/ RVVC. In patients with continued or periodic illness, the prescription of the best antifungal drug is often difficult and requires monitoring of the antifungal sensitivity of the colonizing strain. Moreover, *C. albicans* and *C. glabrata* isolates showed resistance to azole and the caspofungin antifungals without any mutation in the *ERG11* and *FKS1* genes. The present data indicate the possible involvement of other mechanisms, including mutations in the *HS2* region or chitin synthase genes.

## Acknowledgments

The authors received financial support from the Tehran University of Medical Sciences, Tehran, Iran (grant no. 40594) for the current study.

## Authors’ contribution

E.L. and M.F. designed the study. Z.GH performed sample collection. M.E. and E.M. performed antifungal drug susceptibility testing. H.N. performed molecular testing. E.L. and M.F. drafted and performed critical revision of the manuscript. All authors assisted in the edition and revision of the manuscript. 

## Conflicts of interest

The authors declare that they have no competing interests.

## Financial disclosure

The financial support was related to the center for research and training in skin diseases and leprosy.

## Ethical Considerations

This study was approved by the Ethics Committee of Tehran University of Medical Sciences, Iran (ethical code: IR.TUMS.VCR.REC.1397.829).
